# The N-cadherin cytoplasmic domain confers anchorage-independent growth and the loss of contact inhibition

**DOI:** 10.1038/srep15368

**Published:** 2015-10-20

**Authors:** Masayuki Ozawa

**Affiliations:** 1 Department of Biochemistry and Molecular Biology, Graduate School of Medical and Dental Sciences, Kagoshima University, Kagoshima 890-8544, Japan

## Abstract

Tumor growth is characterized by anchorage independence and the loss of contact inhibition. Previously, we showed that either a red fluorescent protein (DsRed)-tagged N-cadherin or E-cadherin cytoplasmic domain (DNCT or DECT) could function as a dominant negative inhibitor by blocking the cell surface localization of endogenous E-cadherin and inducing cell dissociation. Here, we show that expression of DNCT abrogated contact inhibition of proliferation and conferred anchorage-independent growth. DNCT expression induced the relocation of the tumor suppressor Merlin from the cell surface to intracellular compartments. Although DNCT expression induced redistribution of TAZ from the cytoplasm to the nucleus, YAP/TAZ signaling was not activated. An E-cadherin–α-catenin chimera that functions as a β-catenin–independent cell adhesion molecule restored contact inhibition and anchorage-dependency of growth. Addition of the SV40 large T antigen nuclear localization signal reversed the effects of DNCT expression, indicating that DNCT functioned outside of the nucleus.

Cadherins comprise a large family of Ca^2+^-dependent cell–cell adhesion molecules. They interact directly with β-catenin via their cytoplasmic domains. α-Catenin interacts with the cadherins indirectly via interactions with β-catenin and links the cadherin–catenin complex to the actin cytoskeleton through interactions with α-actinin, vinculin, and actin filaments^1^. When the cytoplasmic domain of cadherins were linked directly to α-catenin by genetic engineering technique using cDNA of these proteins, the chimeric proteins mediate strong adhesion independent of β-catenin^2^. β-Catenin also plays a central role in the Wnt signaling pathway. Activation of the β-catenin pathway by Wnt leads to the accumulation of a cytoplasmic pool of β-catenin, which then translocates into the nucleus and binds to transcription factors of the lymphocyte enhancer-binding factor 1 (LEF-1)/T cell factor (TCF) family to regulate expression of β-catenin-LEF–dependent genes, such as cyclin D1 and c-myc^3^,^4^. Dysregulation of the Wnt/β-catenin pathway leads to a constitutively stable and active β-catenin and induces aberrant cell proliferation and malignant transformation^5^.

Increasing cell density arrests epithelial cell proliferation by a process termed contact inhibition. Using MDCK cells, it has been shown that low-density cells proliferate and have higher levels of phospho-ERK1/2 and cyclin D1, and that contact-inhibited high-density cells express low levels of these proteins^6^. Trypsinization of contact-inhibited high-density MDCK cells immediately increases phospho-ERK1/2 and is followed by a transient increase in cyclin D1 levels. Reformation of cell junctions after trypsinization leads to decreases in phospho-ERK1/2 and cyclin D1 levels. These results suggest that, in MDCK cells, contact inhibition of cell proliferation occurs by cell density–dependent regulation of ERK1/2 phosphorylation. Since trypsinization of cells disrupts E-cadherin, and thus E-cadherin–mediated cell–cell adhesion, E-cadherin has been assumed to play critical roles in contact inhibition.

The survival of normal epithelial cells is dependent on their interactions with extracellular matrix, and when deprived of such interactions, they undergo anoikis^7^. Resistance to anoikis is a common feature of many cancers and contributes to tumor progression^8^. Previous reports have implicated β-catenin signaling in the regulation of anoikis. Stable overexpression of β-catenin in MDCK cells has been shown to elevate β-catenin signaling activity, stimulate cell proliferation at high cell densities, promote colony formation in soft agar, and inhibit anoikis^9^. Expression of β-catenin in other cells also prevents anoikis and activates a β-catenin-LEF–responsive reporter gene^10^.

It has been shown that expression of wild-type cadherin inhibits growth of SW480 cells in soft agar. This growth inhibitory activity was mapped to the β-catenin–binding site of the cadherin cytoplasmic domain^11^. Sequestration of β-catenin by cadherin overexpression has been shown to prevent its nuclear translocation and inhibit β-catenin–mediated transcriptional activity^12^. Since the soluble forms of the cytoplasmic tails of N- or E-cadherin have the ability to bind β-catenin, both the membrane-bound and the soluble forms of the cadherin cytoplasmic domains are able to prevent β-catenin signaling^13^,^14^. In addition, E-cadherin inhibits epidermal growth factor (EGF) receptor–mediated growth signaling by β-catenin–independent^15^ or –dependent mechanisms^16^.

The Hippo signaling pathway controls organ size by inhibiting cell proliferation and promoting apoptosis. The pathway stimulates the nuclear exclusion and inactivation of the transcriptional coactivator Yes-associated protein (YAP) and its paralog TAZ (transcriptional activator with PDZ binding motif)^17^. YAP is involved in contact inhibition, as its phosphorylation and nuclear localization are regulated by cell density through the Hippo signaling pathway^18^,^19^,^20^. Overexpression of YAP/TAZ stimulates cell proliferation, reduces cell contact inhibition^21^, and induces anchorage-independent growth in soft agar^22^. Recently, it was shown that E-cadherin, via the Hippo signaling pathway, directly mediates contact inhibition of proliferation by controlling YAP subcellular localization in human MCF10A mammary epithelial cells and MDA-MB-231 cells^23^. A transient reduction in β-catenin levels led to increased YAP nuclear accumulation and decreased YAP phosphorylation at S127^23^. However, studies in the HaCaT human keratinocyte cell line did not support this role of E-cadherin in the activation of the Hippo pathway^24^, so the relationship between E-cadherin and the Hippo signaling pathway remains unresolved.

Previously, we showed that *Discosoma sp.* red fluorescent protein (DsRed)-tagged cadherin cytoplasmic domains expressed in MDCK cells interact with β-catenin, reduce the β-catenin levels associated with endogenous E-cadherin, and inhibit the cell surface localization of endogenous E-cadherin, leading to morphological changes, including the inhibition of desmosome and tight junction formation and a reduction in the mechanical integrity of the epithelial cell sheets^25^. Contrary to previous observations that expression of the cadherin cytoplasmic domains inhibited cell growth by inhibiting β-catenin signaling, we found that expression of the cytoplasmic domains increased cell proliferation. Detailed analysis revealed that expression of the cadherin cytoplasmic domain caused a loss in the contact inhibition of proliferation and conferred resistance to anoikis, while also inhibiting β-catenin and YAP/TAZ signaling. This indicated that these signaling pathways did not mediate DNCT functions. By contrast, DNCT expression induced the relocation of the tumor suppressor Merlin from the cell surface to intracellular compartments. Since Merlin is a critical regulator of contact-dependent inhibition of proliferation and its membrane localization is required for its growth-suppressing function, Merlin relocation may indeed have played a role in DNCT activity.

## Results

### Expression of the N-cadherin cytoplasmic domain increases cell proliferation

**Figure 1:**
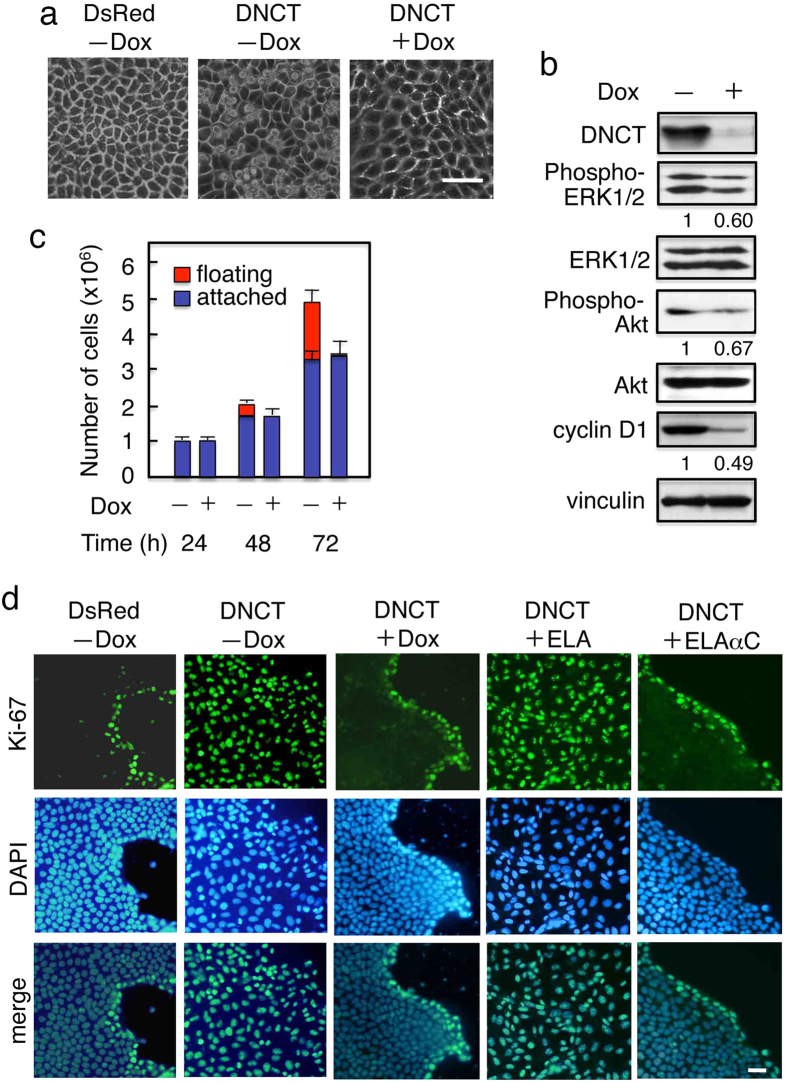
Expression of the DsRed-tagged N-cadherin cytoplasmic domain (DNCT) in MDCK cells increases cell proliferation. Clones showing tet-repressible expression of DNCT or DsRed were isolated and designated DNCT+ or DsRed+, respectively. (**a**) Morphology of cells after 3 d. DsRed+ cells grew in monolayer cultures as epithelial cell sheets with a typical cobblestone morphology, whereas expression of the DNCT protein produced dramatic changes in the DNCT+ cell cultures, including multilayering of the cells, and rounding of cells in the top layer (−Dox). Culturing DNCT+ cells in the presence of Dox (+Dox) completely reversed the morphological changes induced by DNCT expression. (**b**) Immunoblot analysis of confluent cell cultures revealed that p44/42 MAP kinase and Akt were phosphorylated (activated) in DNCT+ (−Dox) cells and that phosphorylation decreased upon Dox treatment (+Dox). DNCT expression also increased levels of cyclin D1. Quantifications obtained by densitometry are indicated below the corresponding panels. DNCT was detected with an anti-FLAG antibody. Vinculin was used as a loading control. (**c**) Growth and detachment of cells from the monolayer. DNCT+ cells were cultured with (+) or without (−) Dox on Transwell plates and cells shed into the medium and on the membranes were counted. Values indicate the mean ± S.E.; n = 3. Approximately 30% of the cells were shed into the medium during 3 d in culture. (**d**) DNCT expression increases the number of Ki-67–positive cells. DsRed+ or DNCT+ cells were cultured for 2 d and were stained with an anti–Ki-67 antibody and DAPI. There was a substantial increase in the number of proliferating (Ki-67–positive) cells in the DNCT+ but not DsRed+ cultures. With respect to the DsRed+ cells, the Ki-67–positive cells were located at the periphery of colonies. Reducing DNCT expression by incubating DNCT+ cells in the presence of Dox (+Dox) decreased the number of proliferating cells, and the Ki-67–positive cells were confined to cells in the periphery. ELAαC (an E-cadherin–α-catenin chimera) expression in DNCT+ cells (DNCT+ELAαC) decreased the number of Ki-67-positive cells, but ELA (a mutant E-cadherin) expression in DNCT+ cells (DNCT+ELA) had no effect. Bars, 25 μm.

We made a chimeric construct (DNCT) comprising a fluorescent protein, DsRed, the N-cadherin cytoplasmic domain (NCT), and a *C*-terminal FLAG tag^25^. The construct was expressed under the control of a Tet-repressible transactivator in an MDCK cell clone T23 expressing the tet repressor (DNCT+ cells). A FLAG-tagged DsRed construct was used as a control (DsRed+ cells). Cells expressing the control DsRed grew in monolayer cultures as epithelial clusters with a typical cobblestone morphology (Fig. 1a). By contrast, expression of the DNCT protein produced dramatic changes in the cultured cells, including multilayering of cells, rounding of cells in the top layer, and detachment of spherical cells from the monolayer (Fig. 1a). Culturing DNCT+ cells in the presence of Dox completely reversed these morphological changes. Immunoblot analysis of DNCT+ T23 cells cultured for 2 d with or without doxycycline showed that DNCT was detected in protein extracts from cells cultured without Dox, but was significantly reduced (~10%) in extracts from cells cultured with Dox (Fig. 1b). During culture of DNCT+ cells in the absence of Dox, we observed a number of floating cells (Fig. 1a, central panel), raising the possibility that DNCT expression induced cell shedding. To address this, cells were grown to confluence in the presence or absence of Dox and the cells shed into the medium were counted. The number of shed cells was markedly elevated in DNCT+ cells cultured in the absence of Dox, as compared to DNCT+ cells cultured in the presence of Dox (Fig. 1c).

To correlate these observations with cell proliferation, we used Ki-67—which is expressed in the nucleus throughout the cell cycle, but is absent in G0^26^—as a proliferation marker. Staining for Ki-67 revealed a substantial increase in the number of proliferating cells in DNCT+ populations but not in DsRed+ populations (Fig. 1d, left two panels). In the case of DsRed+ populations, the Ki-67–positive cells were located at the periphery of colonies. Reducing DNCT expression levels by incubating DNCT+ cells in the presence of Dox decreased the number of proliferating cells, and the Ki-67–positive cells in those cultures were confined to the periphery of colonies (Fig. 1d, central panel). Thus, the expression of DNCT in MDCK cells resulted in the loss of contact inhibition of proliferation.

Contact inhibition is known to involve MAPK and/or PI3K/Akt pathways^6^,^27^, and contact-inhibited confluent cells express low levels of these proteins and as well as cyclin D1. Using MDCK cells, it has been shown that low-density cells proliferate and have higher levels of phospho-ERK1/2 and cyclin D1, and that contact-inhibited high-density cells express low levels of these proteins^6^. Immunoblot analysis using antibodies directed against phosphorylated (activated) ERK1/2 and Akt revealed that these kinases were phosphorylated in DNCT+ cells and that phosphorylation decreased upon Dox treatment (Fig. 1b). Because cyclin D1 is an important element of entry into the cell cycle, we examined cyclin D1 levels and found that they were elevated in cells expressing DNCT (Fig. 1b).

### DNCT expression supports anchorage-independent growth that requires MAP kinase

**Figure 2:**
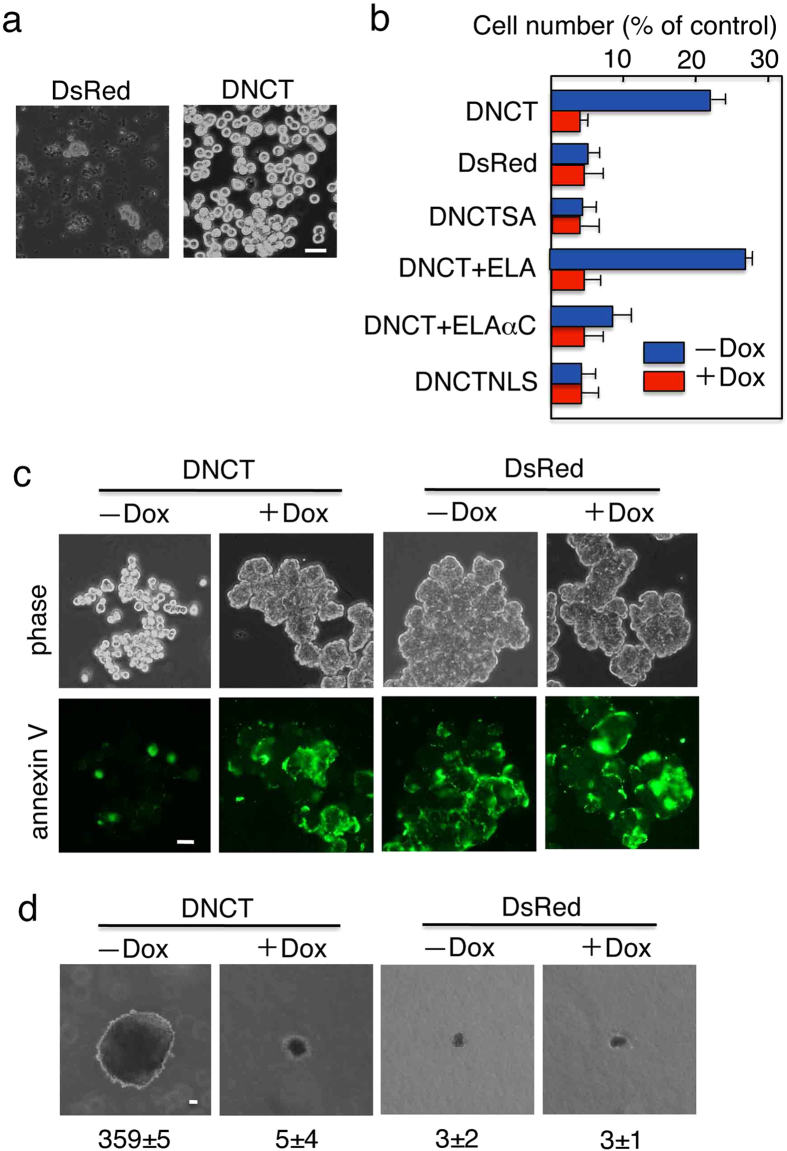
Expression of DNCT in MDCK cells supports anchorage-independent growth. (**a**) Phase contrast micrographs of cells cultured on ultra-low attachment (PrimeSurface^TM^) plates to keep cells in suspension. A significant portion of DsRed+ cells die (dark cells) after 3 d in suspension culture, but DNCT+ cells remain viable (bright cells). (**b**) Quantitation of cell viability after 5 d in suspension culture. Cells expressing DNCT, DsRed, DNCTSA, DNCT and ELA (DNCT+ELA), DNCT and ELAαC chimera (DNCT+ELAαC), or DNCTNLS were cultured in the presence (+) or absence (−) of Dox on normal tissue culture plates (control) or on ultra-low attachment plates and were assayed for their viability using the WST-1 assay. The results are expressed as a percentage of the values obtained with attached (control) cells. Values indicate the mean ± S.E.; n = 3. DNCTSA is a DNCT derivative bearing alanine substitutions of the conserved eight serine residues in the catenin-binding site, which weakens its interactions with β-catenin. DNCTNLS is a DNCT derivative bearing an SV40 NLS signal at its *C*-terminus. (**c**) Cells undergo anoikis in suspension culture; DNCT expression protected against this cell death. Cells were cultured on ultra-low attachment plates for 3 d, and then stained with FITC-annexin V. (**d**) DNCT+ cells show anchorage-independent growth. Cells were cultured in soft agar in the presence (+Dox) or absence (−Dox) of Dox for 21 d. The number of colonies larger than 50 μm in diameter was counted and is shown at the bottom of the pictures. Values indicate the mean ± S.E.; n = 3. Bars, 25 μm.

We noted that suspension culture of DsRed+, but not DNCT+, cells resulted in cell death (Fig. 2a). These findings implied that DNCT could inhibit anoikis resulting from the loss of cell–substrate adhesion. To test this hypothesis, DsRed+ cells and DNCT+ cells with or without Dox treatment were cultured in specialized tissue culture plates, called PrimeSurface^TM^, which prevent cell attachment. Expression of DNCT, but not DsRed alone, promoted the proliferation of cells under suspension culture conditions as measured by the WST-1 assay (Fig. 2b). However, a Dox-induced reduction in DNCT levels decreased the rate of cell proliferation to control (DsRed+) levels. Thus, DNCT expression in T23 MDCK cells protected against anoikis.

To confirm that the floating cells had undergone anoikis, they were stained with FITC-labeled annexin V, an apoptosis marker^28^. After 3 d in suspension culture, a majority (>80%) of DsRed+ cells with or without Dox treatment were stained with annexin V (Fig. 2c). A majority (>70%) of DNCT+ cells with Dox treatment also stained with annexin V. By contrast, a minority (<10%) of DNCT+ cells without Dox treatment were stained with annexin V. Thus, DNCT expression increased the percentage of viable cells in suspension cultures by protecting them from anoikis.

These results raised the possibility that DNCT stimulates anchorage-independent cell growth. Therefore, we investigated whether DNCT expression could mediate the growth of cells in soft agar. The growth of DsRed+ cells with or without Dox treatment and of DNCT+ cells with Dox treatment were slow, and colonies were very small (<50 μm in diameter) even after 21 d in culture (Fig. 2d). The growth of DNCT+ cells in the absence of Dox was fast, and colonies were much larger as compared to DNCT+ cells with Dox treatment.

**Figure 3:**
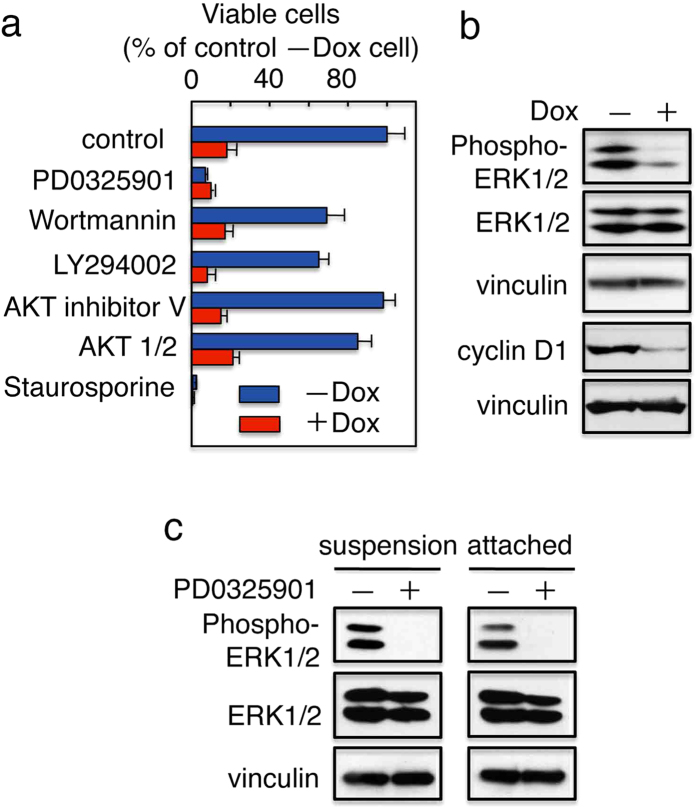
MAP kinase is involved in DNCT-induced protection from anoikis. (**a**) A MEK inhibitor impairs DNCT-induced survival in suspension cultures. A proliferation assay was carried out in the presence of the following chemicals: DMSO (control), PD0325901 (1 μM), Wortmannin (10 μM), LY294002 (25 μM), AKT inhibitor V (30 μM), AKT 1/2 (30 μM), and staurosporine (an apoptosis inducer; 1 μM). DNCT+ cells were cultured on ultra-low attachment plates in the presence (+) or absence (−) of Dox. After 72 h, cells were assayed for their viability using the WST-1 assay. The results were expressed as a percentage of control (DMSO) cells cultured in the absence of Dox (−Dox). Values represent the mean ± S.E.; n = 3. (**b**) The levels of activated MAP kinase and cyclin D1 remained elevated in DNCT+ (−Dox) cells. Cells were cultured on ultra-low attachment plates (in suspension) in the presence (+) or absence (−) of Dox for 24 h. Then, the cells were harvested, and extracts were analyzed by immunoblot using the indicated antibodies. (**c**) The MEK inhibitor PD0325901 blocks phosphorylation of ERK. Cells were cultured on ultra-low attachment plates (suspension) or normal plates (attached) in the presence (+) or absence (−) of PD0325901 (1 μM) for 24 h. After the cells were harvested, extracts were analyzed by immunoblotting using the indicated antibodies. In (**b**,**c**), vinculin was used as a loading control.

DNCT expression led to the activation of MAP kinase and Akt signaling; these signaling pathways have been implicated in the promotion of cell proliferation and survival^29^. Therefore, we tested whether DNCT-mediated protection against anoikis was dependent on these signaling pathways. Indeed, treatment with PD0325901, a potent inhibitor of MAP kinase kinase (MEK), significantly abolished the protective effect of DNCT expression on cells cultured in suspension (Fig. 3a), suggesting that MAP kinase signaling is involved in the rescue of cells from anoikis by DNCT. Treatment with the PI3 kinase inhibitors, Wortmannin and LY294002, slightly inhibited cell growth in the same assay, while treatment with other inhibitors of cell proliferation–related signaling pathways did not significantly influence viability.

Because the MAP kinase pathway appeared to be involved in DNCT-mediated protection against anoikis, we assessed whether MAP kinase itself was activated by DNCT expression in suspension cultures. DNCT+ cells with or without Dox were cultured for 24 h on ultra-low attachment plates. Cells were harvested and lysates subjected to immunoblot analysis using antibodies directed against phosphorylated ERK1/2 and cyclin D1. Although a significant amount of phosphorylated ERK1/2 and cyclin D1 was detected in cells cultured in the absence of Dox, the addition of Dox, which downregulated the expression of DNCT, resulted in a significant reduction in ERK1/2 phosphorylation and cyclin D1 levels (Fig. 3b). These data suggested that DNCT expression in suspension cultures leads to the activation of MAP kinase.

To confirm that MAP kinase signaling is involved in the rescue of cells from anoikis by DNCT, we examined phosphorylation of ERK in cells treated with the MEK inhibitor PD0325901. Western blots revealed that pretreatment of cells with the MEK inhibitor abolished activation of ERK (i.e., phospho-ERK) (Fig. 3c). PD0325901 inhibited the activation of ERK not only in floating cells, but also in attached cells, although it had marginal effect on the growth of the attached cells (data not shown).

### DNCT expression changes the intracellular distribution of Merlin and TAZ

**Figure 4:**
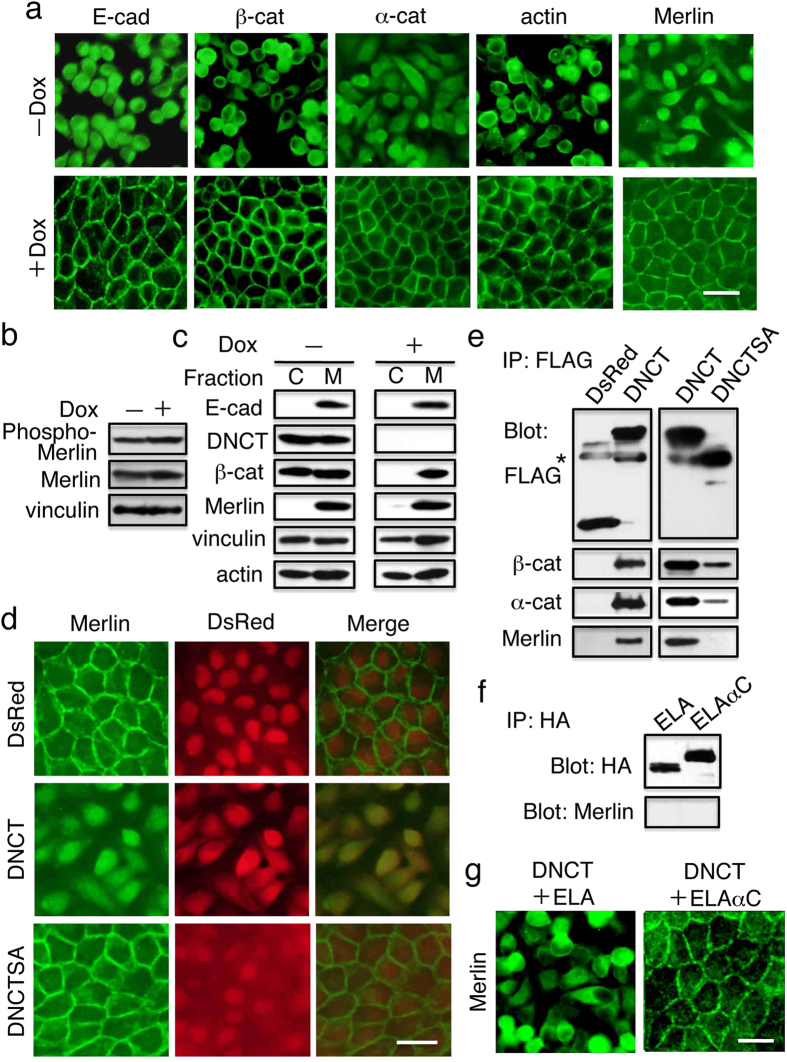
DNCT expression changes the intracellular localization of Merlin. (**a**) Cells were stained with anti–E-cadherin (E-cad), anti–β-catenin (β-cat), anti–α-catenin (α-cat), and anti-Merlin antibodies, and with phalloidin to detect actin. Although DNCT expression led to the intracellular localization of E-cadherin, β-catenin, and Merlin, repression of DNCT expression by Dox addition (+Dox) resulted in the establishment of cadherin-based junctions, including cortical actin filaments and the membrane localization of Merlin. (**b**) Immunoblot detection of Merlin and phospho-Merlin revealed that DNCT expression does not change the amounts of these proteins. Vinculin was used as a loading control. (**c**) Subcellular distribution of E-cadherin, DNCT, β-catenin, and Merlin in DNCT+ cells cultured for 2 d in the presence (+) or absence (−) of Dox. DNCT+ cells were cultured for 2 days in the presence (+) or absence (−) of Dox. The cells were homogenized and centrifuged to obtain cytosolic (C) and particulate (M) fractions. Each fraction was subjected to immunoblot analysis with the indicated antibodies. (**d**) Immunofluorescence staining with an anti-Merlin antibody revealed the co-localization of Merlin with DNCT. By contrast, Merlin did not co-localize with DsRed or with DNCTSA. (**e**) Merlin co-immunoprecipitated with DNCT but not with DsRed or DNCTSA. Immunoprecipitates obtained using an anti-FLAG antibody on lysates from DsRed+, DNCT+, or DNCTSA+ cells were blotted with the indicated antibodies. An asterisk indicates the position of the immunoglobulin heavy chain. (**f**) Merlin did not co-immunoprecipitate with ELA or ELAαC exprssed in DNCT+ cells. HA-tagged ELA or ELAαC were collected using an anti-HA antibody from lysates of DNCT+ELA, or DNCT+ELAαC cells and co-orecipitated materials were blotted with the indicated antibodies. Bars, 25 μm. (**g**) Expression of ELAαC restores intracellular localization of Merlin. Cells were stained with anti-Merlin antibodies. Although expression of ELA did not change the cytoplasmic localization of Merlin, ELAαC expression in DNCT+ cells resulted in membrane localization of Merlin.

Merlin plays an important role in contact inhibition of proliferation^30^,^31^. Upon cell–cell contact, Merlin interacts with the cadherin–catenin complex^32^ and attenuates downstream signaling from EGFR^30^. Merlin is also known to be an upstream regulator of the Hippo signaling pathway and functions through the YAP/TAZ^33^. Importantly, association with the membrane is important for Merlin to function as a growth regulator^34^,^35^. Therefore, we assessed the localization of Merlin in DNCT+ cells with or without of Dox (Fig. 4a). In the presence of Dox, when the cell–cell junctions were established, Merlin was mainly detected at the membrane. By contrast, Merlin was predominantly observed in the cytoplasm in the absence of Dox. Therefore, DNCT expression changed the intracellular localization of Merlin. DNCT did not affect the amounts of phospho-Merlin and Merlin (Fig. 4b); therefore it was not Merlin phosphorylation at S518 that determined its localization. We next examined the membrane versus cytosolic distribution of Merlin, E-cadherin, and β-catenin in DNCT+ cells (Fig. 4c). In DNCT+ cells cultured in the presence of Dox, E-cadherin, β-catenin, and Merlin were primarily found in the membrane (particulate) fraction (Fig. 4c, right panels). When the cells were cultured in the absence of Dox, DNCT was present in both the cytosolic and membrane fractions (Fig. 4c, left panels). Although DNCT expression did not change the distribution of E-cadherin and Merlin, a significant portion (~50%) of β-catenin was present in the cytosolic fraction. Because the membrane fraction also contains cytoskeletons components, including actin and vinculin, it is possible that DNCT may be recovered in this fraction because of its interaction with β-catenin and α-catenin.

Previous experiments showed that Merlin binds directly to α-catenin^32^. Since DNCT binds to β-catenin and forms a complex with α-catenin through β-catenin, it is possible that the complex formation with DNCT changed Merlin distribution. Immunofluorescence staining of DNCT+ and DsRed+ cells revealed that DNCT, but not DsRed, partially colocalized with Merlin (Fig. 4d). Expression of DNCTSA—a DNCT-derivative with alanine substitutions of the conserved eight serine residues in the catenin-binding site, which weakens the interaction with β-catenin^25^—did not impair Merlin membrane localization (Fig. 4d) and did not support anchorage-independent cell growth (Fig. 2b). Immunoprecipitation of lysates made from DNCT+ cells using an anti-Flag antibody followed by immunoblot analysis with an anti-Merlin antibody revealed that Merlin coimmunoprecipitated with DNCT together with α-catenin and β-catenin (Fig. 4e, left panels). Thus, Merlin was a component of the DNCT complex. The same analysis with DNCTSA+ cells revealed that Merlin did not coimmunoprecipitate with DNCTSA (Fig. 4e, right panels).

**Figure 5:**
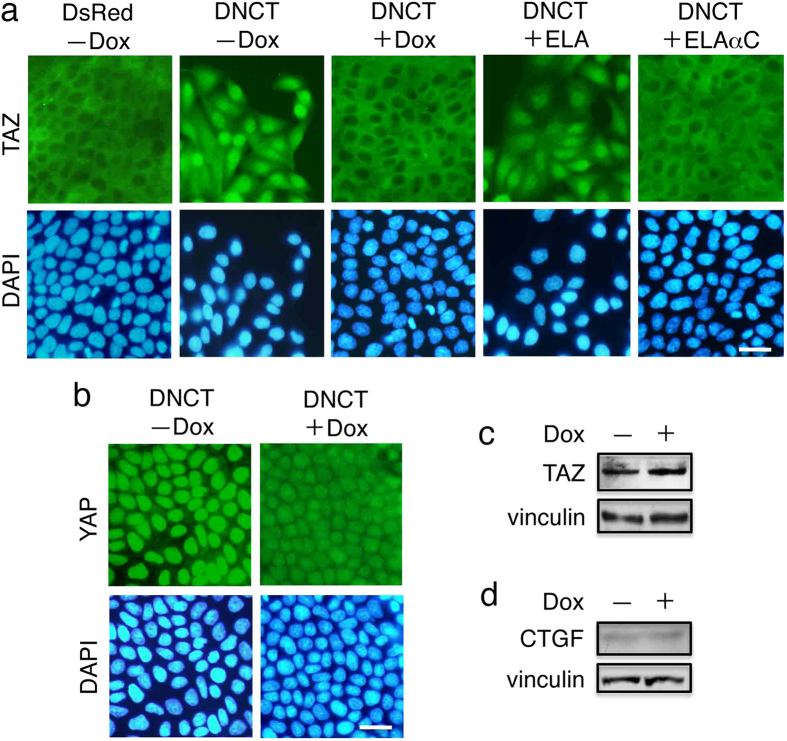
DNCT expression induces the nuclear localization of TAZ. (**a**) Immunofluorescence staining of T23 MDCK cells expressing DsRed, DNCT, or DNCT and ELA (DNCT+ELA), or DNCT and ELAαC (DNCT+ELAαC). Cells were stained with anti-TAZ antibodies and DAPI. Although TAZ is excluded from the nucleus and is predominantly localized in the cytoplasm of DsRed+ cells, TAZ is distributed throughout DNCT+ cells, including a significant portion in the nucleus. Dox-induced reduction in DNCT expression caused the redistribution of TAZ from the nucleus to the cytoplasm. Expression of ELAαC in DNCT+ cells prevents nuclear localization of TAZ induced by DNCT. (**b**) Staining of DNCT+ cells with anti-YAP antibodies and DAPI. Cells were cultured for 2 d in the presence (+) or absence (−) of Dox. Bars, 25 μm. (**c**,**d**) Immunoblot detection of TAZ (**c**) and CTGF (**d**) revealed that the expression of DNCT does not change the amounts of these proteins. Vinculin was used as a loading control.

The Hippo signaling pathway plays an important role in contact inhibition of proliferation^18^,^19^,^36^. In sparse cell cultures, YAP/TAZ is predominantly localized in the nucleus, but in dense cell cultures it is phosphorylated and translocated to the cytoplasm. Accordingly, we examined whether the localization of TAZ was affected by DNCT expression. Although TAZ was excluded from the nucleus and predominantly localized in the cytoplasm of DsRed+ cells, TAZ was distributed throughout DNCT+ cells, with a significant portion localized in the nucleus (Fig. 5a). Interestingly, Dox-induced reduction of DNCT expression led to the redistribution of TAZ from the nucleus to the cytoplasm. Likewise, a significant portion of YAP was localized in the nucleus (Fig. 5b). Dox-induced reduction of DNCT expression led to redistribution of YAP from the nucleus to the cytoplasm, although a portion of YAP remained in the nucleus. DNCT expression did not affect the levels of TAZ (Fig. 5c) or of connective tissue growth factor (CTGF), a well-known YAP/TAZ regulated gene product (Fig. 5d). The latter result suggests that TAZ signaling was not activated despite its nuclear localization.

### DNCT expression inhibits YAP/TAZ and β-catenin signaling

**Figure 6:**
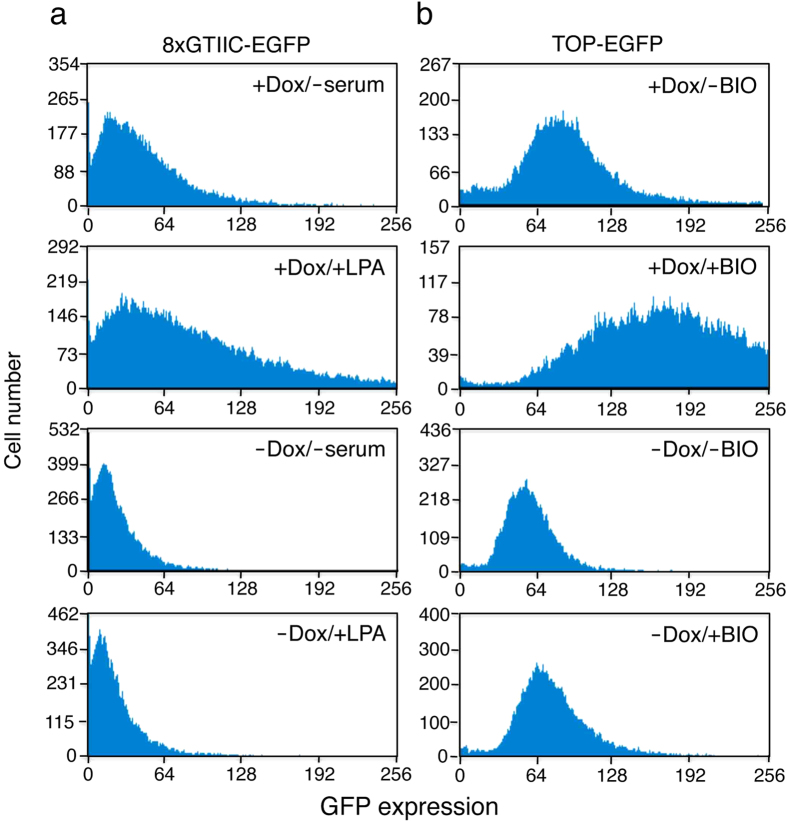
DNCT expression inhibits YAP/TAZ and β-catenin transcriptional activity. A YAP/TAZ-responsive EGFP reporter (8 × GTIIC-EGFP) (**a**) or a β-catenin–responsive EGFP reporter (TOP-EGFP) (**b**) was introduced into DNCT+ cells and stable transfectants were isolated. Cells were cultured for 2 d in the presence (+) or absence (−) of Dox prior to the initiation of the experiments. Then, cells were cultured for 24 h in the presence (+) or absence (−) of LPA (20 μM) or BIO (3 μM) to stimulate YAP/TAZ or β-catenin transcriptional activity, respectively, and were analyzed by flow cytometry.

To determine whether TAZ activity is regulated by DNCT expression, we monitored TAZ transcriptional activity in DNCT+ cells cultured with or without Dox. For this, we used a synthetic YAP/TAZ-responsive EGFP reporter (8×GTIIC-EGFP) as a direct read-out of activity. A similar construct was previously used to successfully monitor TEAD-dependent YAP/TAZ activity^24^. The present construct was introduced into DNCT+ cells and stable transfectants were isolated. 8×GTIIC-EGFP/DNCT cells cultured in the presence of Dox and without serum exhibited low levels of EGFP protein expression as determined by flow cytometry (Fig. 6a, +Dox/-serum). Lysophosphatidic acid (LPA) has recently shown to activate the YAP/TAZ transcription activity^37^, and the addition of LPA to cultures increased expression of the EGFP protein (Fig. 6a, +Dox/+LPA). When the cells were cultured in the absence of Dox, EGFP levels were decreased (Fig. 6a, -Dox/-serum), and YAP/TAZ activity was not activated by the addition of LPA (Fig. 6a, -Dox/+LPA). Consistent with these results, a comparison of the gene expression of DNCT+ cells cultured with or without Dox using an Agilent Whole Canine microarray revealed no change in the expression of CTGF (Table S1). Of the 112 transcripts previously identified as significantly increased by YAP overexpression in NIH 3T3 cells^19^, four were upregulated and three downregulated by DNCT expression; the rest (105 genes) exhibited no change (Table S1 and Table S2). Collectively, these data indicated that although DNCT expression induced the nuclear localization of TAZ and YAP, it was not sufficient to induce the expression target genes.

The Wnt/β-catenin pathway contributes to the self-renewal of stem cells and/or progenitor cells in a variety of tissues^3^. Since β-catenin is a critical player in the Wnt signaling pathway^3^, sequestration of β-catenin by the cadherin cytoplasmic domain has been shown to prevent its nuclear translocation and inhibit β-catenin–mediated transcriptional activity^12^,^13^,^14^. Inhibition of β-catenin signaling by E-cadherin may result in the suppression of cell growth, providing a molecular basis for the adhesion-independent tumor suppressor function of E-cadherin^38^. Although our results are not consistent with the suspected action of the cytoplasmic domain of E- or N-cadherin on the Wnt/β-catenin pathway, we investigated the effect of DNCT expression on β-catenin transcriptional activity. We performed a reporter assay using a construct expressing EGFP under the control of tandem repeats of a LEF-1/TCF binding site (TOP-EGFP)^39^. The same construct was previously used to successfully monitor LEF-1–dependent β-catenin activity^40^. The construct was introduced into DNCT+ cells and stable transfectants were isolated. TOP-EGFP/DNCT cells cultured in the presence of Dox exhibited low levels of EGFP protein expression as determined by flow cytometry (Fig. 6b, +Dox/-BIO). BIO (6-Bromoindirubin-3’-oxime)—which inhibits GSKβ3, a kinase that phosphorylates and renders β-catenin susceptible to proteosomal degradation, and thus stabilizes β-catenin—activates β-catenin transcription activity^41^. The addition of BIO to the cultures increased the expression of the EGFP protein (Fig. 6b, +Dox/+BIO). When the cells were cultured in the absence of Dox, EGFP expression levels were reduced (Fig. 6b, -Dox/-BIO), and the β-catenin transcriptional activity was not activated by the addition of BIO (Fig. 6b, -Dox/+BIO). These data indicated that DNCT expression did not activate, but instead inhibited β-catenin–mediated transcriptional activity, consistent with previous reports^13^,^14^,^42^.

### Expression of an E-cadherin–α-catenin chimeric molecule that functions as a β-catenin–independent cell adhesion molecule restores contact inhibition of and anchorage-dependency of growth

To obtain more insight into the role of the cadherin-catenin complex, we expressed a mutant E-cadherin (ELA) or an E-cadherin–α-catenin chimera (ELAαC) in DNCT+ cells. ELA is a mutant E-cadherin in which the two leucine residues at positions 587 and 588 were replaced with two alanine residues (LA substitution). This substitution improves the cell surface localization of E-cadherin when β-catenin is not available^25^. Although the reason is not clear, the introduction of the LA substitution in EαC increased efficiency of the cell surface localization of the fusion protein in epithelial cells such as MDCK cells. ELAαC, a chimeric molecule of E-cadherin and α-catenin, does not interact with β-catenin, but still functions as a cell adhesion molecule^25^. Although expression of ELA in DNCT+ cells did not change the number of Ki-67-positive cells, expression of ELAαC decreased the number of Ki-67-positive cells (Fig. 1d, right two panels). When these cells were cultured under suspension conditions, DNCT+ cells expressing ELAαC exhibited a significant reduction in cell proliferation, whereas expression of ELA had no effect (Fig. 2b). Thus, ELAαC expression in DNCT+ cells restored contact inhibition and anchorage-dependency of growth.

ELAαC has the *C*-terminal one-third of the α-catenin polypeptide (residues 612–906). The Merlin binding site of α-catenin has been mapped to its N-terminal VH1 domain^32^. Consistent with this, immunoprecipitaion of ELAαC with anti-HA antibody, followed by immunoblotting to detect Merlin, revealed that ELAαC does not bind with Merlin (Fig. 4f). When DNCT+ cells expressing ELAαC were stained with anti-Merlin antibody, a significant portion of Merlin was detected at the membrane, whereas in DNCT+ expressing ELA, Merlin remained predominantly in the cytoplasm (Fig. 4g). It remains unclear how ELAαC, an E-cadherin–α-catenin chimeric molecule that lacks the Merlin-binding site, can induce membrane localization of Merlin. Clearly, however, the restoration or establishment of cell–cell adhesion, rather than a simple molecular interaction between α-catenin and Merlin, is critical.

Next, we investigated whether the localization of TAZ was affected by ELAαC expression in DNCT+ cells. Although a significant portion of TAZ was localized in the nucleus in DNCT+ cells, its distribution was not affected by the expression of ELA. By contrast, ELAαC expression changed the distribution of TAZ, causing it to be excluded from the nucleus and predominantly localized in the cytoplasm (Fig. 5a, right two panels).

### DNCT acts in the cytoplasm

**Figure 7:**
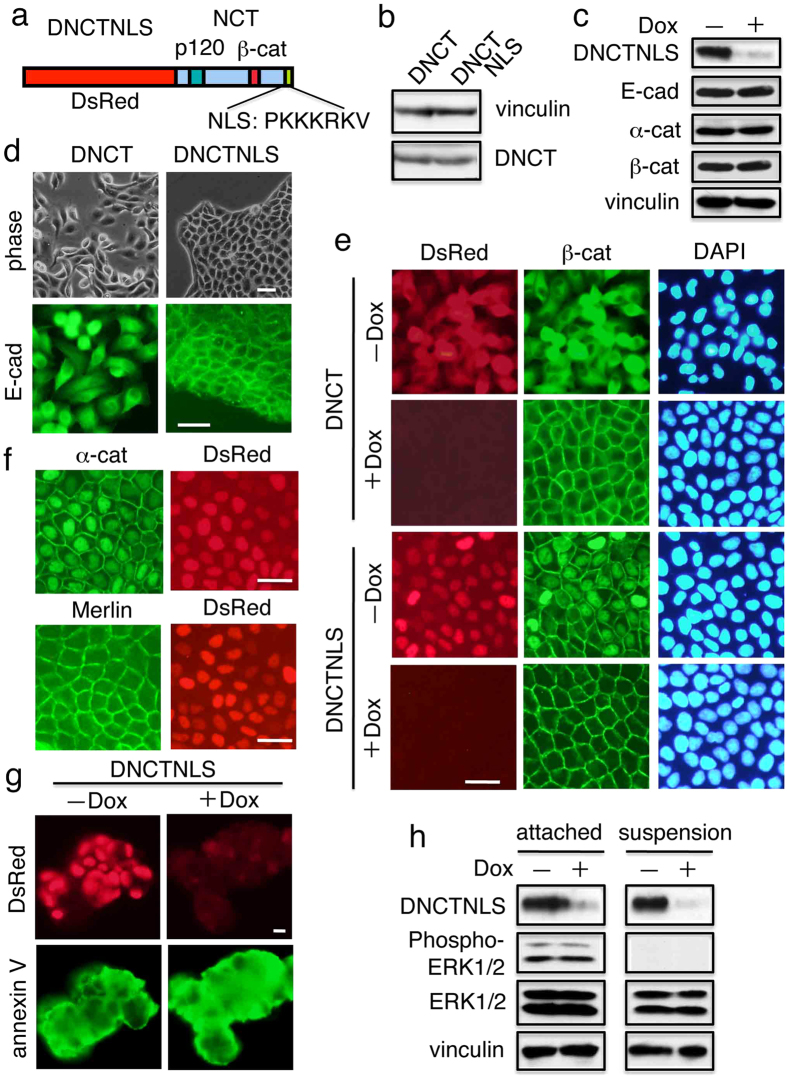
Addition of NLS inactivates the potential of DNCT. (**a**) Schematic representation of DNCTNLS, a derivative of DNCT with the SV40 large T antigen at the *C*-terminus. DNCTNLS and DNCT both have a FLAG tag at their *C*-termini. (**b**) Immunoblot analysis revealed that comparable amounts of DNCT and DNCTNLS were produced by the stable transfectants. The blots were probed with anti-vinculin (a loading control) and anti-FLAG antibodies. (**c**) Immunoblot analysis revealed that the levels of E-cadherin, α-catenin, and β-catenin do not change upon DNCTNLS expression. (**d**) DNCTNLS does not impair the cell surface transport of endogenous E-cadherin. E-cadherin was detected with DECMA-1. (**e**) DNCTNLS induces the nuclear accumulation of β-catenin, but does not deplete β-catenin from the cell surface. (**f**) As in the case of β-catenin, a significant portion of α-catenin colocalized with DNCTNLS in the nucleus, but Merlin remained on the cell-surface membrane. Thus, the interaction of Merlin with α-catenin takes place in the cytoplasm, but not in the nucleus. Bars, 25 μm. (**g**) DNCTNLS does not inhibit anoikis. Cells were cultured in suspension in the presence (+) or absence (−) of Dox for 3 d, and then stained with FITC-annexin V. (**h**) DNCTNLS failed to enhance ERK signaling. DNCTNLS+ cells were cultured on normal plates (attached) or on ultra-low attachment plates (suspension) in the presence (+) or absence (−) of Dox. Then, the cells were harvested, and extracts were analyzed by immunoblot using the indicated antibodies. In (**c**,**h**), vinculin was used as a loading control.

It has been shown that the PS1-dependent ε-cleavage product of N-cadherin, the soluble N-cadherin cytoplasmic domain, binds the transcription factor CBP (CREB-binding protein), promotes its proteasomal degradation, and inhibits CRE-dependent transactivation^43^. Thus, the domain functions as a potent repressor of CBP/CREB (cyclic AMP responsive element–binding protein)-mediated transcription. Intriguingly and in contrast to those reports, the soluble cytoplasmic N-cadherin domain has also been shown to translocate to the nucleus and enhance β-catenin signaling^44^,^45^. Furthermore, nuclear expression of the E-cadherin cytoplasmic domain tagged with NLS has been shown to suppress staurosporine-induced apoptosis^46^. Therefore, we asked whether nuclear localization of DNCT facilitated the inhibition of anoikis. To this end, we made a construct encoding DNCT with the SV40 large T antigen NLS at its *C*-terminus (DNCTNLS; Fig. 7a), and introduced it into T23 MDCK cells. Although immunoblot analysis revealed that comparable amounts of DNCT and DNCTNLS were produced by the stable transfectants (Fig. 7b), DNCTNLS expression did not change the levels of E-cadherin, α-catenin, or β-catenin (Fig. 7c), nor did not impair the cell-surface transport of endogenous E-cadherin (Fig. 7d), or deplete β-catenin from the cell surface (Fig. 7e). As in the case of β-catenin, a significant fraction of α-catenin colocalized with DNCTNLS in the nucleus, whereas Merlin remained on the cell surface (Fig. 7f). More importantly, after 3 d in suspension culture, a majority (>80%) of DNCTNLS+ cells with or without Dox treatment were stained by annexin V (Fig. 7g). Thus, the addition of NLS to DNCT inactivated its ability to suppress anoikis. Consistent with these observations, DNCTNLS failed to enhance ERK signaling under both normal (attached) and suspension conditions (Fig. 7h).

## Discussion

Epithelial cells exhibit contact inhibition arising from cell–cell interactions. Contact inhibition ensures that epithelial cells will stop proliferating once they have reached confluence. Most human cancer cells are refractory to contact inhibition. As a consequence, they are able to continue proliferating in spite of interactions with neighboring cells. Many established cancer cell lines also exhibit growth *in vitro* that is impervious to contact inhibition, and often display anchorage-independent growth in soft agar. The loss of contact inhibition and the gain of anchorage-independent growth are hallmarks of cancer cells *in vitro*^47^.

Cadherins mediate Ca^2+^-dependent cell adhesion and cell junction formation, and their cytoplasmic domains are associated with various catenins that mediate cytoskeletal association and signaling^48^. E-cadherin is expressed in epithelial cells and intercellular homophilic binding of E-cadherin leads to the formation of the epithelial junctional complex and a tight polarized cell layer^48^. Loss of E-cadherin expression through genetic or epigenetic alterations promotes tumor progression and metastasis^49^,^50^. On the other hand, overexpression of E-cadherin in cancer cells impedes tumor progression and invasion^50^,^51^. This inhibition has a dual origin, due both to E-cadherin’s adhesive function at the cell surface, which physically blocks the movement of cells and facilitates other cell–cell interactions, and to its inhibition of β-catenin signaling and other growth signaling pathways^11^,^16^,^42^. Thus, cadherin-mediated cell–cell adhesion is thought to play an important role in contact inhibition. However, the mechanisms underlying the contact inhibition of proliferation remain poorly understood.

In this paper, we showed that the expression of DNCT (the soluble N-cadherin cytoplasmic domain) in MDCK cells promoted cell proliferation on plastic dishes, in suspension cultures, and in soft agar and conferred resistance to anoikis. DNCT+ cells had higher levels of phospho-Erk1/2 and cyclin D1. Reducing DNCT expression by the addition of Dox abolished cell proliferation and reduced the levels of phospho-Erk1/2 and cyclin D1, demonstrating that DNCT expression had led to those changes. Addition of PD0325901, an inhibitor of MEK, a kinase that phosphorylates ERK1/2, significantly reduced the proliferation of cells in suspension cultures. DNCT interacted with the tumor suppressor Merlin through associations with α- and β-catenin. This interaction changed the distribution of Merlin from the cell surface, where it regulates mitogenic pathways, to the cytoplasm, presumably abolishing its activity. Since Merlin is a critical mediator of contact-dependent inhibition of proliferation and a suppressor of anchorage-independent growth, inactivation of Merlin may have led to the observed increases in phospho-MAPK and cyclin D1, which in turn caused the loss of contact inhibition and the acquisition of anoikis-resistance in DNCT+ cells. We do not know the reason why E-cadherin–α-catenin fusion protein lacking the Merlin-binding site restored the membrane localization of Merlin, contact inhibition and anchorage-dependency of growth. It has been reported that a mutant Merlin lacking the N-terminal 17 amino acids cannot associate with α-catenin but could still interact with Par3^32^. We previously found that the expression of the chimera in DECT+ cells restored cell–cell adhesion and junction formation, including tight junction^25^. Merlin can likely assemble multiple different complexes even within a given cell. The assembly of such complexes may responsible for the membrane localization of Merlin, contact inhibition and anchorage-dependency of growth in cells expressing the fusion protein.

The Wnt/β-catenin pathway contributes to the self-renewal of stem cells and/or progenitor cells in a variety of tissues. Thus, deregulated activation of this pathway has been implicated in a number of cancers^3^. Since β-catenin is a critical player in the Wnt signal transduction pathway^3^, sequestration of β-catenin by the cadherin cytoplasmic domain has been shown to prevent its nuclear translocation and inhibit β-catenin–mediated transcriptional activity^12^,^13^,^14^. Inhibition of β-catenin signaling by E-cadherin may result in suppression of cell growth, providing a molecular basis for the adhesion-independent tumor suppressor function of E-cadherin^38^. Consistent with these previous reports, DNCT expression inhibited β-catenin signaling. Therefore, β-catenin signaling was not involved in the loss of contact inhibition and the acquisition of anoikis resistance in DNCT+ cells.

Stable overexpression of β-catenin in MDCK cells has been shown to elevate β-catenin signaling activity, stimulate cell proliferation at high cell densities, promote colony formation in soft agar, and inhibit anoikis^9^. Expression of β-catenin in other cell types also prevents anoikis, activates a β-catenin-LEF–responsive reporter gene, and induces endogenous cyclin D1 (a target of β-catenin signaling) expression^10^. Resistance to anoikis correlated with the increased phosphorylation of MAP kinase, and was abolished by treatment with the MAP kinase pathway inhibitor, PD98059. Thus, MAP kinase also played a critical role in the anoikis resistance conferred by β-catenin expression. The mechanism by which β-catenin can activate p42/44 MAP kinase is not yet defined.

Although DNCT expression induced the nuclear accumulation of TAZ, YAP/TAZ signaling was not activated. We do not know why TAZ was not active in spite of its nuclear localization. There are several reports indicating a tight correlation between the Wnt/β-catenin pathway and the Hippo/YAP/TAZ pathway. Namely, YAP1 is regulated by the Wnt/β-catenin pathway^52^, the Hippo/YAP/TAZ pathway regulates β-catenin signaling^53^,^54^,^55^, and YAP1 is essential for β-catenin–dependent cancers^56^. One possible explanation is that YAP/TAZ activity requires β-catenin. DNCT expression may reduce the availability of β-catenin for YAP/TAZ signaling and repress YAP/TAZ activity. If that is the case, this would be the first experiment showing that YAP/TAZ activity required β-catenin.

## Methods

### cDNA construction

The vectors, (pC-DNCT), containing the *N*-terminally DsRed-tagged and *C*-terminally FLAG-tagged N-cadherin cytoplasmic domain construct, and (pU-DNCT), the expression vector for DNCT under the control of the Tet-repressible transactivator^57^, were described previously^25^. The vector containing DNCTSA, a DNCT-derivative with alanine substitutions of the conserved eight serine residues in the catenin-binding site, was constructed in the same way using pC-NSAHA^58^ as a PCR template. The expression vector for DNCTNLS was constructed by inserting double stranded oligonucleotides, (CCGGTCCAAAAAAGAAGAGAAAGGTAGAT and ATCTACCTTTCTCTTCTTTTTTGGA), encoding SV40 NLS (PKKKRKV) between the N-cadherin cytoplasmic domain and the FLAG tag. The vectors, pC-ELAHA and pC-ELAαCHA, encoding E-cadherin derivatives, were described previously^25^.

The 8×GTIIC-luciferase plasmid containing 8 TEAD binding sites (ACATTCCA)^20^ was obtained from Addgene (Cambridge, MA, USA). To construct the reporter plasmid 8×GTIIC-EGFP, the cDNA encoding luciferase from the 8×GTIIC-luciferase plasmid was removed by digestion with NcoI and EcoNI, and replaced with EGFP cDNA isolated by digestion with NcoI and NotI. To construct the reporter plasmid, pTOP-EGFP, the promoter/enhancer regions of the expression vector pEGFPN1 (Clontech, Mountain View, CA, USA) were removed by cleavage with XhoI and AseI, and replaced with an XbaI-fragment from the TCF-activatable plasmid pTOPFLASH, containing three TCF-binding elements in front of the c-fos minimal promoter^39^. All PCR products were sequenced and cloned into expression vectors. pCAGGShyg, which confers hygromycin resistance, has been described^25^. The pCAG/bsr-7 vector containing a blasticidin-resistance gene^25^ was provided by Masahiro Sato (Kagoshima University).

### Cells and transfections

Cells were transfected with expression vectors and selected using either hygromycin (300 μg/ml) or blasticidin (8 μg/ml). Stable transfectants expressing the anticipated proteins were identified by fluorescence microscopy and immunoblot, and were isolated as previously described^25^. At least three independent clones were selected for each construct to ensure that any observed effects were not due to phenotypic variability introduced by clonal selection. MDCK T23 cells^59^,^60^ (provided by W. James Nelson, Stanford University) were transfected with pU-DNCT (15 μg) and pCAGGGShyg (1.5 μg) using calcium phosphate, and hygromycin-resistant clones were isolated and examined for DNCT expression as described above. For repression of DNCT expression, cells were cultured in the presence of doxycycline (Dox, 20 ng/ml) for the indicated period. Since DNCT MDCK T23 cells already had G418-, puromycin-, and hygromycin-resistance, we used a pCAG/bsr-7 vector with a blasticidin-resistance gene to isolate DNCT+ cells expressing 8×GTIIC-EGFP or TOP-EGFP.

### Antibodies

DECMA-1, a mAb recognizing the extracellular domain of E-cadherin (provided by Rolf Kemler, Max-Planck Institute for Immunobiology) was used to detect E-cadherin. A mouse mAb recognizing FLAG (DYKDDDDK) was purchased from Wako Pure Chemical Industries, Ltd. (Osaka, Japan). Mouse mAbs recognizing β-catenin and TAZ were purchased from BD Biosciences (Lexington, KY, USA); mAbs recognizing vinculin, actin, and pan-cadherin were obtained from Sigma-Aldrich Japan (Tokyo, Japan). Anti-Ki-67 was from Dako Japan (Tokyo, Japan). The rabbit mAbs, p44/42 MAPK (Erk1/2), phospho-p44/42 MAPK (Erk1/2) (Thr202/Tyr204), Akt, phospho-Akt (Ser473), phospho-Merlin (Ser518), YAP, and the rabbit polyclonal antibody, phospho-Merlin (Ser518) were from Cell Signaling Technology (Danvers, MA, USA). Rabbit anti-Merlin (NF2) and anti-CTGF (connective tissue growth factor) were from Santa Cruz Biotechnology (Dallas, TX, USA). Rabbit anti-cyclin D1 was obtained from MBL Japan (Nagoya, Japan). All secondary antibodies were obtained from Jackson ImmunoResearch Laboratories, Inc. (West Grove, PA, USA).

### Immunoprecipitation and immunoblotting

Immunoprecipitation and immunoblot analyses were carried out as described^25^. In brief, cells (2 × 10^6^) were lysed in a buffer of 25 mM Tris-HCl pH 7.4, 1% Triton X-100, 2 mM EDTA, 10 mM sodium pyrophosphate, 10 mM NaF, 1 mM Na_3_VO_4_, 1 mM PMSF, 10 μg/ml leupeptin, and 25 μg/ml aprotinin. Proteins of interest were enriched by using mAbs that had been preabsorbed to protein G-Sepharose. Western blot signals were quantitated using the ImageJ software, and relative intensities were calculated after normalization against the corresponding vinculin signals.

### Cell fractionation

Cells were washed with ice-cold phosphate-buffered saline (PBS) and collected in 1 ml of ice-cold PBS containing protease inhibitors, and then passed 10 times through a 22-gauge needle. The homogenized cells were centrifuged at 14,000 × *g* for 30 min at 4 °C. Proteins of the supernatant (cytosolic protein) and the pellet (membrane protein) fractions were subjected to immunoblot analysis as described above.

### Fluorescence microscopy

Immunofluorescence labeling of cells was performed as described^25^. In brief, cells were fixed with 3% paraformaldehyde in PBS for 20 min at room temperature. Cells were permeabilized with 0.1% Triton X-100, and then incubated with primary and secondary antibodies. Cells were analyzed using an Olympus fluorescence microscope (Tokyo, Japan) equipped with a CD72 camera (Olympus).

### Flow cytometry

Cells were incubated for 2 d with or without Dox, trypsinized, and then 1 × 10^5^ cells were plated and cultured on 35 mm dishes for 24 h under the conditions specified. EGFP expression in DNCT+ cells that were stably transfected with the reporter plasmid pTOP-EGFP and stimulated by the addition of LPA or BIO was analyzed on a Beckman Coulter CyAn ADP analyzer (Beckman Coulter Japan, Tokyo, Japan).

### Quantification of cell shedding

Cells were seeded at a density of 5 × 10^5^ cells/well on Corning Transwell 3412 plates (Costar, Cambridge, MA, USA). At the times indicated, media were removed, cells were trypsinized, and the cells in the media and on the membranes were counted using a hemocytometer.

### Anoikis assay

Cells were seeded onto ultra-low attachment plates for suspension culture. After 3 d, cells were harvested, incubated with FITC-annexin V (1 pg/ml; MBL) for 10 min, and the percentage of stained cells was determined using a fluorescence microscope.

### WST-1 cell proliferation assay

A WST-1 assay for cell proliferation, which measures succinate-tetrazolium reductase activity, was performed according to the manufacturer’s instructions (Dojindo Co. Kumamoto, Japan). Briefly, cells (2 × 10^4^) were suspended in 1 ml of DME containing 10% FBS and cultured in 24-well tissue culture plates or on ultra-low attachment plates for 5 d. Cell proliferation reagent (100 μl per well) was added and cells were incubated at 37 °C for 10–30 min. The number of viable cells was obtained by measuring the optical density (OD) at 450 nm.

### Soft agar colony assay

Cells (5 × 10^3^) were suspended in 1.5 ml of DME containing 10% FBS and 0.35% agar (Difeo Laboratories, Detroit, MI, USA), and plated into the same medium containing 0.5% agar on a 6-well plate. The wells were covered with 1 ml of culture medium. Cells were incubated with or without Dox (20 ng/ml) for 21 d at 37 °C. Colonies were stained with 0.05% crystal violet for visualization.

### Gene expression microarray and data analysis

DNCT+ cells were cultured for 2 d with or without Dox, and then total RNA was prepared using TRIzol Reagent (Life Technologies Japan, Tokyo, Japan) and purified using the SV Total RNA Isolation System (Promega KK, Tokyo, Japan). cRNA was amplified and labeled using a Quick Amp Labeling Kit (Agilent Technologies, Santa Clara, CA, USA) and hybridized to a 44 K Agilent 60-mer oligomicroarray (Canine Oligo Microarray Kit; Agilent Technologies). The hybridized microarray slides were scanned using an Agilent scanner. The relative hybridization intensities and background hybridization values were calculated using Agilent Feature Extraction Software (version 9.5.1.1). Microarray data analysis was supported by Cell Innovator (Fukuoka, Japan).

## Additional Information

**How to cite this article**: Ozawa, M. The N-cadherin cytoplasmic domain confers anchorage-independent growth and the loss of contact inhibition. *Sci. Rep.* **5**, 15368; doi: 10.1038/srep15368 (2015).

## Supplementary Material

Supplementary Table S1 & S2
