# Supplementary material for: The N-cadherin cytoplasmic domain confers anchorage-independent growth and the loss of contact inhibition
Source: Sci Rep. 2015 Oct 20;5:15368. doi: 10.1038/srep15368 (PMC4612716; doi:10.1038/srep15368)
Supplement: Supplementary Table S1 & S2 [file srep15368-s1.pdf]

Supplementary Information

The N-cadherin cytoplasmic domain confers anchorage-independent growth and the loss of contact inhibition

Masayuki Ozawa*

Table S1. Genes Not Upregulated by DNCT Expression

ABCC3	**CASC5**	CXCL2	HIST1H2BD	MAF	PNRC1	SPC25
**ABCC9**	**CCNB1**	DCN	HIST3H2A	**METTL7A**	**PRC1**	ST6GAL1
AMPD3	**CCNB2**	**DIAPH3**	HLA-E	MICALL2	PRELP	TCF4
**ANLN**	CCND1	DTL	HMGA2	**MKI67**	**PRR11**	**TK1**
AQP3	**CDC2**	EGR1	**HMMR**	**NCAPG**	PRSS23	TMEPAI
**ARHGAP29**	**CDC20**	ETV5	INHBA	NCOA7	REEP1	TNFAIP3
**ASPM**	**CDCA2**	**FANCB**	**INSIG1**	**NDC80**	RELB	**TOP2A**
AXL	**CDKN3**	FBXO32	**IQGAP3**	NFKBIZ	RFFL	TOP3B
**BUB1**	CENPA	FEN1	**KIF2C**	**NUF2**	RHOU	TP53INP1
C1orf25	**CENPE**	FGF2	**KIF4A**	NUP62	RYR1	**TPX2**
C1orf25	**CENPF**	FLRT2	**KIF11**	**NUSAP1**	SAA2	TRIB3
C5orf15	**CEP55**	FOXO3	**KIF14**	**PDGFRL**	SERPINA3	**TTK**
**C6orf173**	**CIT**	FST	**KIF15**	PDK4	**SLIT2**	**UBE2C**
**C13ORF3**	**CKAP2L**	GBP2	**KIF20A**	PLA2G4A	SMC2	WISP2
C20orf82	CTGF	H2BFS	KLF9	PLAC8	SOD2	ZNF292

Transcripts for 105 genes shown in this table were previously identified as significantly upregulated upon YAP overexpression in NIH 3T3 cells[19]. The 45 genes in boldface font denote transcripts in this list whose expression is significantly upregulated by matrix stiffness[60].

Table S2. Changes in relative expression levels of YAP-inducible genes upon DNCT expression in MDCK cells.

Gene symbol	GenBank Accession	+Dox→-Dox
PTGS2	NM_001003354	+4.90
LCN2	DN873779	+4.61
DUSP6	XM_539711	+3.70
GPNMB	XM_858105	+2.68
CYR61	Not assigned	-4.96
SERPINE1	NM_001197095	-6.57
SEPP1	NM_001115118	- 7.17

Gene expression profiles of DNCT+ in the presence and absence of Dox were compared using Agilent Whole Canine Genome microarrays. Data are presented as the intensities of signals in DNCT+ cells cultured in the presence of Dox, relative to the corresponding signals from DNCT+ cells cultured in the absence of Dox.

PTGS2, prostaglandin-endoperoxide synthase 2 (prostaglandin G/H synthase and cyclooxygenase) (PTGS2), LCN2, lipocalin 2, DUSP6: dual specificity phosphatase 6, GPNMB: P glycoprotein (transmembrane) nm; CYR61, cysteine-rich, angiogenic inducer, 61; SERPINE1, serpin peptidase inhibitor, clade E (nexin, plasminogen activator inhibitor type 1), member 1 (SERPINE1); SEPP1, lupus familiaris selenoprotein P, plasma, 1.
